# Case Report: Bloodstream infection with *Nocardia cyriacigeorgica* in a patient with diffuse large B-cell lymphoma

**DOI:** 10.3389/fmed.2025.1595190

**Published:** 2025-09-11

**Authors:** Ning Du, Bingqing Xu, Hongmin Wang, Haochun Tang, Jianping Li, Jun Meng, Dan You

**Affiliations:** ^1^Department of Pharmacy, National Cancer Center/National Clinical Research Center for Cancer/Cancer Hospital & Shenzhen Hospital, Chinese Academy of Medical Sciences and Peking Union Medical College, Shenzhen, Guangdong, China; ^2^Department of Pharmacy, Qiqihar Hospital of Traditional Chinese Medicine, Qiqihar, Heilongjiang, China; ^3^Department of Hematology, The First Hospital of Qiqihar, Qiqihar, Heilongjiang, China; ^4^Department of Pharmacy, The First Hospital of Qiqihar, Qiqihar, Heilongjiang, China

**Keywords:** *Nocardia cyriacigeorgica*, nocardiosis, bloodstream infection, septic shock, diffuse large B-cell lymphoma

## Abstract

*Nocardia* bacteremia is a rare but life-threatening opportunistic infection associated with high mortality rates. Early diagnosis and prompt initiation of appropriate treatment are crucial for improving patient outcomes. In our study, we report a patient with diffuse large B-cell lymhoma who unfortunately had a bloodstream infection with *Nocardia cyriacigeorgica*. The patient presented with characteristic clinical manifestations of septic shock, including persistent high-grade fever, hypotension, and acute renal insufficiency. Despite receiving empirical treatment with imipenem for 6 days following admission, the patient remained febrile with temperatures reaching 39°C. Subsequent blood cultures identified *N. cyriacigeorgica*, prompting a modification of the antimicrobial regimen to a triple-drug combination of imipenem, linezolid, and trimethoprim-sulfamethoxazole (TMP-SMX). Following the initiation of therapy, the patient demonstrated significant clinical improvement and was subsequently discharged. Based on this clinical experience and the severity of nocardial infections, we recommend combination therapy with two to three antimicrobial agents for the management of *Nocardia* infections.

## 1 Introduction

*Nocardia* are aerobic Gram-positive bacteria and are widely distributed in soil, dust, decaying vegetation, and aquatic environments (1). *Nocardia* infection typically occurs in immunocompromised hosts, such as solid-organ or hematopoietic stem cell transplant patients, malignancies, human immunodeficiency virus infection, primary immune deficiencies and those receiving long-term treatment with steroids or immunosuppressants (2). *Nocardia* are usually transmitted to humans through inhalation or inoculation into the skin (3, 4). Thus, nocardial infections mainly manifest as pulmonary, or have skin involvement (5, 6). However, the bacteria can disseminate hematogenously to distant organs or spread to adjacent tissues by contiguous invasion (6). *Nocardia* bacteremia is rare, accounting for approximately 1.3%–7.7% of patients with *Nocardia* infections (7, 8). It is reported that the mortality rate among patients with *Nocardia* bacteremia is as high as 40% (9).

Cases of *Nocardia* bacteremia have been documented in patients with advanced T-cell lymphoma, pediatric acute lymphoblastic leukemia, and malignant lymphoma (10–12). To date, no literature reports have described *Nocardia* bacteremia in diffuse large B-cell lymphoma patients. In this study, we report a case of a patient with diffuse large B-cell lymphoma, who survived *Nocardia* bacteremia, providing valuable experience for clinical reference.

## 2 Case presentation

**FIGURE 1:**
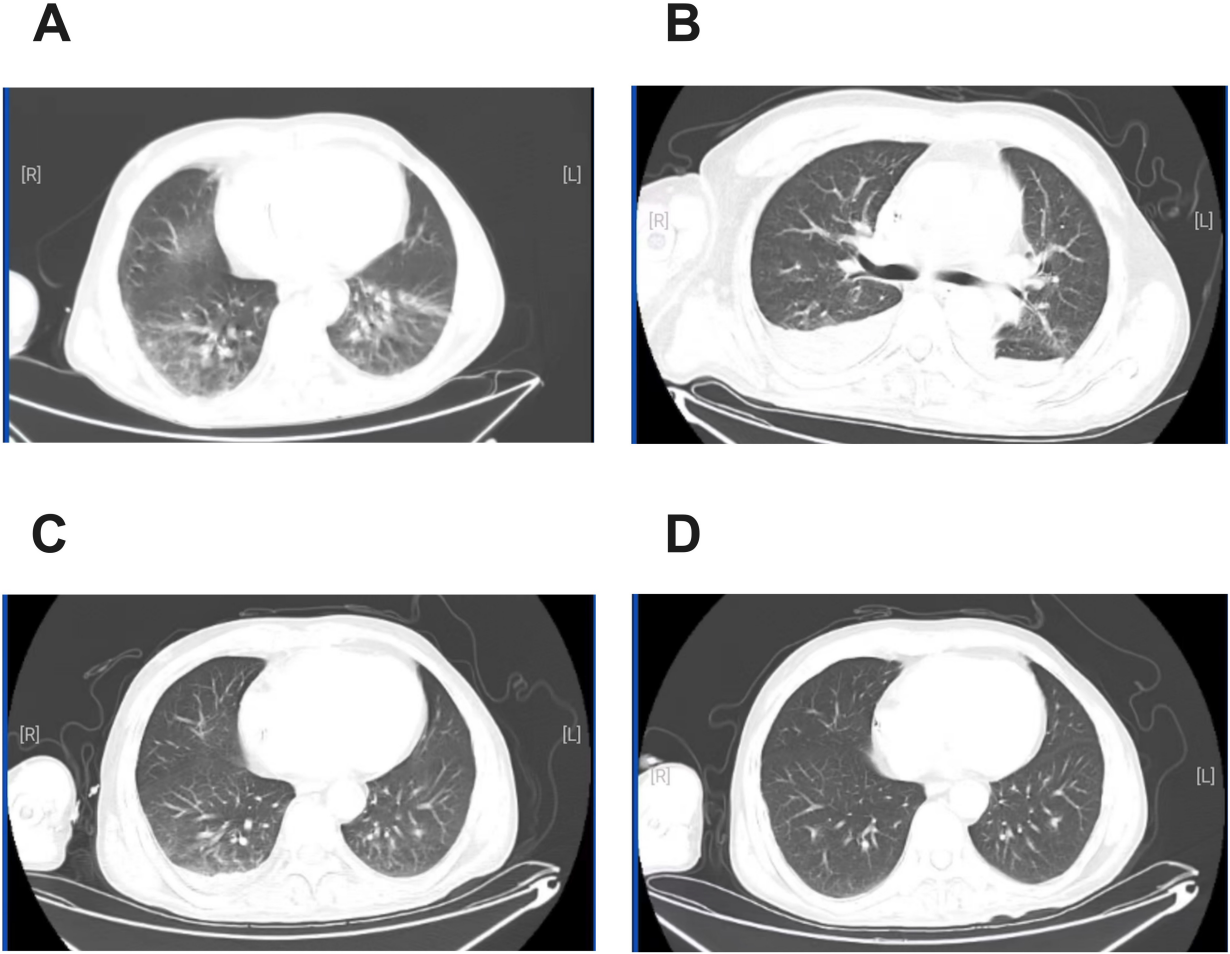
CT of the chest. **(A)** Admission. **(B)** After 6 days of treatment with imipenem. **(C)** After 16 days of treatment with imipenem, linezolid and TMP-SMX **(D)**. After 23 days of treatment with imipenem, linezolid and TMP-SMX.

**FIGURE 2:**
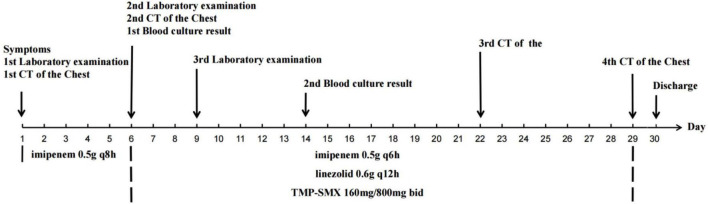
Clinical course timeline.

The patient was a 55-year-old man weighing 56.5 kg. BMI was 18.66 kg/m^2^. He was diagnosed with diffuse large B-cell lymphoma five months previously and was undergoing regular chemotherapy. He was admitted to our hospital with fever for 3 days. His vital signs were as follows: temperature 39°C, blood pressure 87/50 mmHg, pulse 108 beats/min, and respiratory rate 26 breaths/min with an oxygen saturation of 95% on room air. Laboratory examination showed white blood cell count (WBC): 3.9 × 10^9^/L, the proportion of neutrophils was 76.9%, C-reactive protein (CRP): 189.3 mg/L, Procalcitonin (PCT): 4.46 ng/mL, creatinine: 366.5 μmol/L, eGFR: 16.12 mL/min (Table 1). Chest Computed Tomography (CT) was obtained, showing streaks, nodules and patchy abnormal shadows in both lungs (Figure 1A). The patient was diagnosed with septic shock. Blood and sputum cultures were obtained and the patient was initially empirically treated with imipenem (0.5 g q8h iv) for 5 days. On day 6 of treatment (D6), the temperature was 39°C, WBC count was 6.88 × 10^9^/L, the proportion of neutrophils was 92.6%, the CRP level was 63.17 mg/L, PCT decreased to 2.098 ng/mL, the creatinine was 156.7 μmol/L (Table 1). The follow-up CT scan revealed reticular shadows, nodules, patchy infiltrates, and areas of consolidation in both lungs with ill-defined margins; atelectasis in the lower lobes of both lungs, and bilateral pleural effusion (Figure 1B). Blood aerobic culture was positive on the automatic blood culture system. The identification was performed by MALDI-TOF MS (Autof MS 1000), confirming the isolate as *Nocardia cyriacigeorgica.* The confidence score was 99.9%. Due to challenges associated with antimicrobial stability and the fastidious growth characteristics of *Nocardia spp.*, most clinical laboratories in China do not routinely perform susceptibility testing. The antibiotic regimen was changed to imipenem (0.5 g q6h iv), linezolid (0.6 g q12h iv) and trimethoprim-sulfamethoxazole (TMP-SMX) (160 mg/800 mg bid) for 23 days (13). After counseling on risks of undiagnosed intracranial pathology, the patient declined brain imaging studies due to absence of symptoms suggestive of intracranial infection (13, 14). After 3 days of treatment (D9), the patient’s temperature was around 38°C. Neutrophil%, CRP, PCT and creatinine decreased (Table 1). On D14, the patient’s clinical condition improved. A repeat blood culture was negative. On D22, chest CT showed significant reduction in the lesion area (Figure 1C). The patient’s temperature was normal. On D29, the result of chest CT was bilateral pulmonary nodules (Figure 1D). The patient was afebrile. The patient was discharged from the hospital on the 30th day after the admission. There were no adverse drug reactions observed during treatment. After discharge, the patient was asked to continue oral TMP-SMX and repeat chest CT imaging regularly (clinical course summarized in Figure 2).

**TABLE 1:** Blood test results.

Laboratory tests	D1	D6	D9	D14	D22
WBC (×10^9^/L)	3.9	6.88	6.97	7.01	6.89
Proportion of neutrophils (%)	76.9	92.6	83.6	79.6	70.7
PCT (ng/ml)	4.46	2.098	1.005	0.296	0.16
CRP (mg/ml)	189.3	63.17	52.85	36.14	18.51
Creatinine (umol/L)	366.5	156.7	138.2	163	166
eGFR (mL/min/1.73 m^2^)	16.12	44.45	51.74	42	41

WBC, white blood cell count; PCT, procalcitonin; CRP, C-reactive protein.

## 3 Discussion

According to the *List of Prokaryotic Names with Standing in Nomenclature*, 120 *Nocardia* species have been described currently, and up to 54 *Nocardia* species have been shown to cause disease in humans (15–17). The most commonly identified species in humans include *N. nova, N. brasiliensis, N. farcinica, N. cyriacigeorgica, N. brevicatena* and *N. abscessus* (18). In China, *N. farcinica* is the most common species (19). Nocardiosis is confirmed by identifying the organism through culture, which usually takes several days to several weeks (20). Furthermore, it can also be diagnosed by molecular testing of samples obtained from a suspected site of infection (20, 21). Whole genome sequencing (WGS) is a high-throughput method (22). It has become an invaluable tool for doctors. WGS can provide an unprecedented amount of information and significantly streamlining diagnostics, particularly for slow-growing pathogens (23). WGS enables the sequencing of *Mycobacterium tuberculosis* and the identification of drug-resistant tuberculosis (DR-TB) profiles by detecting known mutations (24, 25). Detection of *Nocardia* species in human samples has been achieved through WGS (22, 26, 27). WGS can also be used to reveal the genomic diversity, taxonomic classification, and evolutionary relationships of the genus *Nocardia* (28).

*Nocardia* bacteremia is a rare occurrence with a high mortality rate of 40%, and is a severe form of disseminated nocardiosis (9). Most patients with bacteremia have pulmonary infection and are immunocompromised, which is consistent with this patient (9). Early diagnosis and treatment of *Nocardia* bacteremia are critically important to prognosis. At present, the choice of antimicrobials is based on cumulative retrospective experience (29). For severe infection with *Nocardia*, initial empirical treatment is recommended with a combination of two or three antimicrobials (13, 14). Although TMP-SMX has been used as part of first-line therapy for nocardiosis, not all *Nocardia* species are susceptible; thus, antibiotic sensitivity testing is still recommended when feasible (30, 31). Amikacin, imipenem, moxifloxacin, minocycline, linezolid, tigecycline and dapsone also have activity against *Nocardia in vitro* (32). Different *Nocardia* species have varying susceptibility patterns. *N. cyriacigeorgica* is usually susceptible to TMP-SMX, imipenem, ceftriaxone, and amikacin but resistant to amoxicillin-clavulanic acid (7, 33). However, the susceptibility to carbapenems varies between geographical regions and among different *Nocardia* species (7, 18, 33). The optimal duration of antimicrobial treatment for severe disease has not been determined and usually depends on the patient’s immune status.

In our case, the patient was initially treated with imipenem monotherapy, but unfortunately, the treatment was unsuccessful. According to the results of blood culture, the patient was subsequently treated with TMP-SMX, imipenem, and linezolid for 23 days. Following discharge, the patient continued to receive oral TMP-SMX for 12 months.

The limitations of this study should be mentioned. Further prospective and well-designed clinical trials are required, especially with large samples, to further evaluate the therapeutic effects of the combination of TMP-SMX, imipenem, and linezolid.

## 4 Conclusion

*Nocardia* bacteremia has a high mortality rate, and early diagnosis and treatment directly affect the prognosis. Our report suggests that, for *Nocardia* bacteremia, severe pulmonary infection, or disseminated disease, a combination of two or three antimicrobials is essential.

## Data Availability

The original contributions presented in this study are included in this article/supplementary material, further inquiries can be directed to the corresponding authors.
